# Obesity and Roux-en-Y gastric bypass drive changes in miR-31 and miR-215 expression in the human rectal mucosa

**DOI:** 10.1038/s41366-021-01005-y

**Published:** 2021-10-29

**Authors:** Stella Panagio Breininger, Laura Sabater, Fiona Caroline Malcomson, Sorena Afshar, Jelena Mann, John Cummings Mathers

**Affiliations:** 1 Human Nutrition Research Centre, Population Health Sciences Institute, Newcastle University, Newcastle upon Tyne, NE2 4HH UK; 2 Biosciences Institute, Newcastle University, Newcastle upon Tyne, NE2 4HH UK; 3 North Cumbria University Hospital NHS Trust, Cumberland Infirmary, Newtown Road, Carlisle, CA2 7HY UK

**Keywords:** Genetics, Cancer

## Abstract

**Background/Objectives:**

Obesity increases colorectal cancer (CRC) risk. However, the effects of weight loss on CRC risk are unclear. Epigenetic mechanisms involving microRNAs that lead to dysregulated gene expression may mediate the effects of obesity and weight loss on CRC risk. We examined the effects of obesity and weight loss following Roux-en-Y gastric bypass (RYGB) on microRNA expression in the human rectal mucosa.

**Subjects/Methods:**

We collected rectal mucosal biopsies from obese patients (*n* = 22) listed for RYGB and age- and sex-matched healthy non-obese Controls (*n* = 20), at baseline and six months post-surgery. We quantified microRNA expression in rectal mucosal biopsies using Next Generation Sequencing and bioinformatics analysis to investigate the likely functional consequences of these epigenetic changes.

**Results:**

Compared with non-obese individuals, obese individuals showed differential expression of 112 microRNAs (p < 0.05). At six-months post-RYGB, when mean body mass had fallen by 27 kg, 60 microRNAs were differentially expressed, compared with baseline (*p* < 0.05). The expression of 36 microRNAs differed significantly between both i) obese and non-obese individuals and ii) obese individuals pre- and post-RYGB. Quantitative polymerase chain reaction (qPCR) demonstrated that expression of miR-31 and miR-215 was significantly (*p* < 0.05) higher, 143-fold and 15-fold respectively, in obese than in non-obese individuals. Weight loss, following RYGB, reduced expression of miR-31 and miR-215 to levels comparable with Controls. These differentially expressed microRNAs are implicated in pathways linked with inflammation, obesity and cancer.

**Conclusion:**

Our findings show, for the first time, that obesity is associated with dysregulated microRNA expression in the human rectal mucosa. Further, surgically-induced weight loss may normalise microRNA expression in this tissue.

## Introduction

The prevalence of obesity has doubled in more than 70 countries since 1980 and the incidence continues to rise [1]. Obesity increases colorectal cancer (CRC) risk. Data from 56 observational studies involving over seven million people, including 93 812 CRC cases, showed that higher Body Mass Index (BMI) was associated with increased CRC risk in both retrospective and prospective studies; each 5 kg/m^2^ increment in BMI was associated with an 18% increase in risk [2]. In addition, excess adiposity is a risk factor for colorectal adenomas [3], precursors of CRC, indicating that increased adiposity may act at an early stage in colorectal tumourigenesis [4]. Compared with lifestyle-based interventions, bariatric surgery results in much greater and more sustained body weight loss, and lowers the risk of several [5, 6]. The effect of intentional weight loss, following bariatric surgery, on CRC risk remains uncertain with some studies reporting increased, and others decreased, risk [4].

Epigenetic mechanisms involving microRNAs (miRNAs) that lead to dysregulated gene expression may mediate the effects of obesity and weight loss on CRC risk. MiRNAs are small, single-stranded non-coding RNA molecules, ~22 nucleotides long, that are expressed in all nucleated cells. MiRNAs regulate gene expression at the transcriptional, or post-transcriptional, level by binding in a sequence-specific manner to the complementary region in the 3ʹ–untranslated mRNA region which subsequently regulates translation of the mRNA to protein [7, 8]. Aberrant patterns of miRNA expression are involved in the initiation and progression of oncogenesis, including CRC, through their role as tumour suppressor genes (TSG) and oncogenes [8]. Furthermore, abnormal expression of miRNAs is also observed in obesity where they play roles in adipocyte differentiation, proliferation, clonal expansion and insulin resistance [9–11]. However, most of this evidence has been obtained from measurements made in easily accessible tissue, such as blood and adipose tissue, and there are no comparable data on miRNA expression from measurements made in the human colorectal epithelium.

We hypothesised that miRNAs are (i) aberrantly expressed in obese individuals compared with healthy non-obese people and (ii) modulated by significant weight loss following Roux-en-Y gastric bypass (RYGB)––the most widely used type of bariatric surgery. For the first time, we present quantitative measures of global miRNA expression in the rectal mucosa of adults with obesity before and after RYGB. We reveal a distinct epigenetic modulation by changes in adiposity that may characterise altered CRC risk. We show that miRNA expression profiles are altered with excess obesity and following RYGB, indicating that the epigenetic landscape of the human rectal mucosa is plastic and susceptible to changes in adiposity that may drive tumorigenesis.

## Materials/subjects and methods

### BOCABS participant cohort

Ethical approval was obtained from the NRES Committee, North East - Newcastle and North Tyneside 2 (13/NE/0204) and the project was recorded on the ISRCTN register under the following code: ISRCTN95459522. Informed written consent for participation in the BOCABS study was obtained. Rectal mucosal biopsies were collected from 22 age and sex-matched Caucasian patients at baseline and at six months post-RYGB and from 20 age- and sex-matched healthy non-obese Controls at North Tyneside General Hospital (exclusion criteria are described in [ref. 12]). Anthropometric measurements, including weight, BMI, body fat percentage (using bioimpedance Tanita TBF-300MA Body composition analyser) and waist and hip circumferences, were taken in the morning after at least six hours fast.

### Collection of blood and rectal mucosal biopsies

Blood samples were collected for evaluation of fasting plasma glucose (FPG), high sensitivity C-reactive protein (hs-CRP), haemoglobin A1c (hbA1c), insulin, homeostasis model assessment insulin resistance and leptin. In the absence of bowel preparation, the rectum was examined by rigid sigmoidoscopy and rectal mucosal pinch biopsies (2.2 mm) were collected in a circumferential manner at 10 cm from the anal margin, immediately snap-frozen in liquid nitrogen and stored at −80 °C until laboratory analysis.

### MiRNA expression analysis identified by Next Generation Sequencing (NGS)

RNA was extracted from frozen rectal mucosal biopsies (miRNeasy Mini Kit, catalog number/ ID: 217004, Qiagen) and RNA integrity was assessed by spectrophotometry. Small RNA libraries were generated (NEBNext® Multiplex Small RNA Library Prep Set for Illumina® (Set 1 (catalog number/ ID: E7300S) and Set 2 (catalog number/ ID: E7580S), New England BioLabs)) and sequenced using the Illumina MiSeq sequencer (MiSeq Reagent Kit v3). Raw fastq reads obtained from sample library sequencing were analysed using the Chimira pipeline [13] (https://www.ebi.ac.uk/research/enright/software/chimira) to trim (adaptor sequence “AGATCGGAAGAGC”), and for size selection, mapping and quality control analysis. MiRNA count data were normalised and corrected for batch effects, using DESeq2 package for R (v 3.01) [14] (see Table S1 and S2 for raw and normalised miRNA counts, respectively, and Table S3 for mean raw and normalised miRNA counts). Differential expression analysis was performed using DESeq2 package for R. MiRNAs with a log2 fold-change (logFC) >1 and an adjusted *p* value < 0.05 were classified as differentially expressed. Further analysis was performed using a custom R script to generate volcano plots and heatmap matrices, gplots package for R (v 3.01) (https://CRAN.R-project.org/package=gplots).

### Ingenuity pathway analysis of identified miRNA targets

MiRNAs with expression levels that were considered significantly different (p-adjusted < 0.05 and absolute logFC >1) were entered into the Ingenuity Pathway Analysis (IPA) tools (http://www.ingenuity.com) for functional annotation.

### Validation of miRNA expression by qPCR

A panel of the top four up- and top four downregulated miRNAs, with the greatest and significant fold change between pre- and post-RYGB, identified by NGS, was selected for validation by quantitative polymerase chain reaction (qPCR) using validated primers. Extracted RNA from the rectal mucosal biopsies was reverse-transcribed into cDNA and qPCR was performed using miScript primer assays on a Step One Plus qPCR machine running StepOne 7500 Software version 2.0.6 (Applied Biosystems). Two reference/ housekeeping genes, namely *SNORD68* and *RNU6*, were quantified alongside each target miRNA and the ΔCt method was applied to determine miRNA expression.

### Statistical analysis

Statistical analyses were performed in IBM® SPSS® Statistics Version 21 and significance was set at *p* < 0.05. Data were not normally distributed (determined by the Shapiro-Wilk test) and non-parametric tests were applied. The Mann–Whitney *U* test was used to compare differences between obese and non-obese Controls. To examine the effects of weight loss in obese participants before and after RYGB, the Wilcoxon-signed-rank test was applied.

## Results

### Characteristics of participants in the biomarkers of colorectal cancer after bariatric surgery (BOCABS) study

To analyse changes in miRNA expression patterns in adults with obesity and following RYGB, we collected rectal mucosal biopsies from 20 non-obese Controls and from 22 matched obese patients pre- and at six months post-RYGB, recruited to the BOCABS Study [15]. Obese participants had a mean age of 47 years (range 30.9–65.2 years) and there were more females (*n* = 18) than males (*n* = 4) (Table 1). At baseline, mean BMI was 42.4 kg/m^2^ and body fat was 47.6% and these dropped significantly to 31.3 kg/m^2^ and 36.1%, respectively, at six months after RYGB. This weight, and fat, loss was associated with remission from type two diabetes in two (out of four) participants. As expected, weight loss improved systemic markers of insulin resistance and inflammation, which are predictors of reduced CRC risk [15, 16]. Non-smoking was a stringent inclusion criterion for bariatric surgery but, at six months follow-up, one participant had started smoking daily and 11 occasionally. The non-obese Control group (*n* = 20) had a mean age of 46 (range 21–61) years with more females (*n* = 12) than males (*n* = 8), mean BMI 25.4 kg/m^2^ (range 20–30 kg/m^2^) and mean body fat 30.3% (range 21.2–36.2%). The majority of participants (*n* = 12) had never smoked, a third (*n* = 6) reported being current smokers (smoked daily/ occasionally) and two were ex-smokers.

**Table 1:** Clinical characteristics of BOCABS Study participants pre- and post-RYGB and of non-obese Controls.

	Obese pre-RYGB (*n* = 22)	Obese post-RYGB (*n* = 22)	Non-obese controls (*n* = 20)	*P* value; pre-RYGB vs Controls	*P* value; pre- vs post-RYGB
Age (years)*	47 (1.2)	–	46 (2.6)	0.720	–
Sex *N* (%)					
Female	18 (82)	–	12 (60)	0.175^b^	–
Male	4 (18)	–	8 (40)		
Smoking *N*					
Daily	0	1	5	**0.002**^b^	**<0.001**^‡^
Occasional	0	11	1		
Ex-smoker	11	9	2		
Never smoked	10	0	12		
Missing data	1	1	0		
NSAID administration *N*					
Yes	10	1	5	0.209^b^	**0.004**^c^
No	12	21	15		
Cholecystectomy *N*					
Yes	4	0	2	0.665^b^	–
No	18	22	18		
Diabetes Mellitus *N*					
Yes	4	2	0	0.679^b^	1.0^c^
No	18	19	9		
Missing data	0	1	11		
Weight (kg)^a^	114.8 (3.7)	87.7 (3.5)	71.8 (2.8)	**<0.001**	**<0.001**
BMI (kg/m^2^)^a^	41.7 (1.4)	31.8 (1.1)	25.4 (0.5)	**<0.001**	**<0.001**
Body fat (%)^a^	47.6 (1.0)	36.5 (1.5)	30.3 (1.3)	**<0.001**	**<0.001**
Waist: Hip ratio^a^	0.92 (0.0)	0.87 (0.0)	0.86 (0.0)	**0.039**	**0.001**
Female	0.89 (0.0)	0.84 (0.0)	0.82 (0.0)	**0.010**	**0.007**
Male	1.07 (0.0)	0.99 (0.0)	0.93 (0.0)	**0.001**	0.067
FPG (mmol/L)^a^	5.8 (0.4)	4.9 (0.4)	4.5 (0.1)	**0.003**^¶^	**0.001**^‡^
hsCRP (mg/L)^a^	5.5 (0.9)	1.6 (0.4)	3.6 (1.2)	0.190^¶^	**<0.001**^‡^
HbA1c (mmol/-mol)^a^	42.2 (2.9)	38.5 (2.4)	36.6 (0.8)	0.194^¶^	**0.001**^‡^
Insulin (pmol/L)^a^	117.1 (19.6)	54.3 (7.8)	64.6 (10.6)	**0.025**^¶^	**0.001**^‡^
HOMA-IR^a^	2.1 (0.4)	1.0 (0.1)	1.2 (0.2)	**0.014**^¶^	**<0.001**^‡^
Leptin (ng/mL)^a^	62.5 (14.3)	15.6 (3.9)	11.2 (3.2)	**0.002**^¶^	**0.003**^‡^

An unpaired *t*-test was used for comparisons between pre-RYGB and non-obese Controls unless otherwise stated as ^¶^ Mann–Whitney *U* test for non-parametric data

A paired *t*-test was used for comparison between pre- and post-RYGB unless otherwise stated as ^‡^Wilcoxon signed rank test for non-parametric data

*FPG* Fasting Plasma Glucose, *hsCRP* high sensitivity C-reactive protein, *HbA1c* haemoglobin A1c, *HOMA-IR* homeostasis model assessment insulin resistance.

^a^Data presented as Mean (SEM)

^b^Fisher’s exact test

^c^Related sample McNemar test

### Genome-wide miRNA expression patterns are altered in obesity and reversed by RYGB

NGS, followed by IPA and qPCR was performed to characterise global and differential miRNA expression patterns in the rectal mucosa of i) matched groups of obese and non-obese (Controls) adults, ii) obese patients before and after RYGB and iii) non-obese (Controls) adults and adults post-RYGB.

### Effects of adiposity on genome-wide miRNA expression patterns

**Fig. 1:**
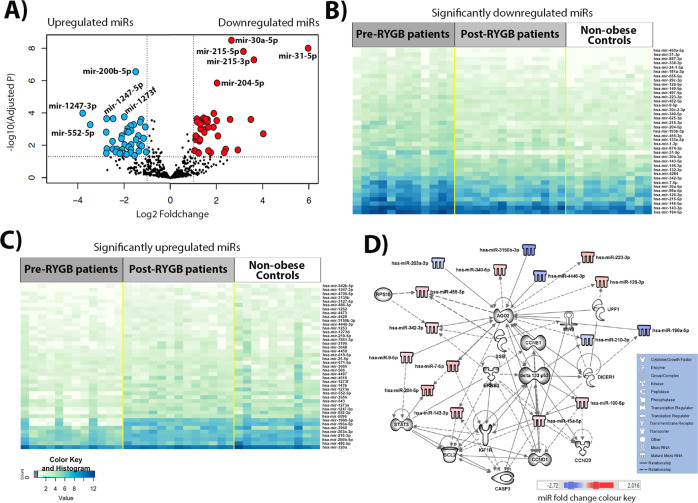
Differential miRNA expression in obese individuals before RYGB compared with non-obese Controls, identified by NGS. **A** Volcano plot illustrating 43 significantly upregulated miRNAs (red dots, top 5 miRNAs are annotated: miR-31-5p, miR-215-3p, miR-215-5p, miR-30a-5p and miR-204-5p) and 39 downregulated miRNAs (blue dots, top five are annotated: miR-1273f, miR-200b-5p, miR-1247-5p, miR-552-5p and miR-1247-3p) with fold change >1 (**B**) Heatmap of significantly up-regulated miRNAs, for which expression differed significantly between the obese individuals pre-RYGB and the non-obese Controls. **C** Heatmap of significantly downregulated miRNAs, for which expression differed significantly between the obese individuals pre-RYGB and the non-obese Controls. **D** IPA network of miRNAs for which expression differed by logFC >1 in the obese pre-RYGB and non-obese Controls and their predicted target molecules.

NGS identified a total of 1 654 miRNAs in rectal mucosal biopsies of all participants. When compared with non-obese Controls, obese individuals before RYGB showed significant differential expression of a total of 112 miRNAs (logFC ranged from −5.97 to 3.78), of which 49 and 63 were significantly up- and downregulated, respectively (Fig. 1A and Table S4). Figure 1B and C show heatmaps for expression of the most significant up- and downregulated miRNAs, respectively, in obese individuals pre- and post-RYGB and in non-obese Controls (blue boxes indicate higher number of miRNA counts and white lower number of miRNA counts). These heatmaps show the level of significant miRNA expression for all samples clustered by group. A differential expression pattern between the non-obese Controls and obese individuals pre-RYGB is evident. Using the 82 miRNAs for which expression differed by absolute logFC >1 between obese individuals before RYGB and non-obese Controls, IPA predicted a network that involved 31 molecules (Fig. 1D). These include key molecules required for miRNA biogenesis, *AGO2* and *DICER1*, that show multiple interactions with the miRNA and other molecules in this network. Notably, *AGO2* is targeted by 10 network members (*UPF1*, *CCNE1*, *DELTA133P53*, miR-9, miR-9-5p, miR-100-5p, miR-196a-5p, miR-203-3p, miR-3150b-3p and miR-4446-3p), targets 7 network members (miR-126-3p, miR-143-3p, miR-204-5p, miR-210-3p, miR-223-3p, miR-340-5p, miR-455-3p) and is associated with a further 3 molecules in this network (*DICER1*, miR-7-5p, miR-3342-3p). In turn, *DICER1* is targeted by *DELTA133P53*, targets miR-9, miR-15a-5p, miR-196a-5p, and is associated with *AGO2* and *UPF1*. Regulatory molecules including *DELTA133P53*, cyclin E (*CCNE1*) and *STAT3*, which play roles in CRC development, are also actors in the network [17–22]. *DELTA133P53* is targeted by *ERBB2*, targets 6 network members (*DICER1, CCND3, CASP3, AGO2*, miR-100-5p and miR-210-3p) and is associated with a further three molecules (*IGF1R, BCL2* and miR-15a-5p) in this network. The interplay between the miRNAs and other molecules in this network is predicted to be involved in cancer, gastrointestinal (GI) disease, inflammatory disease and response, cellular growth and proliferation, tumour morphology, cell-to-cell signalling and interaction, cell death and survival, cellular growth and proliferation (Figure S1 and Table S5).

### Effects of RYGB on genome-wide miRNA expression in the rectal mucosa

**Fig. 2:**
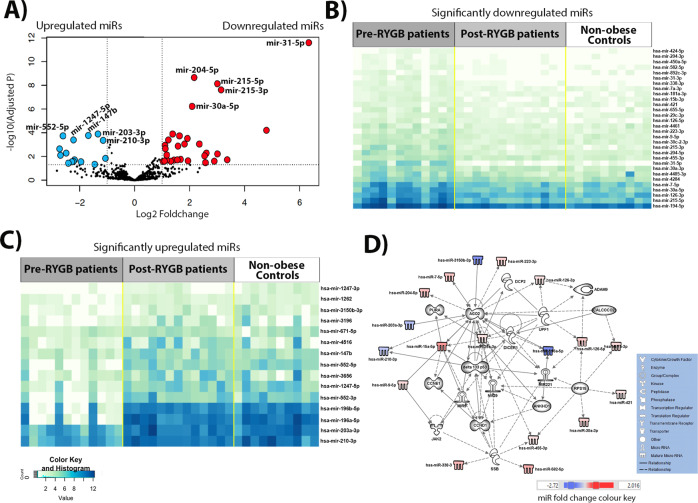
Differential miRNA expression in obese individuals pre- compared with post-RYGB, identified by NGS. **A** Volcano plot illustrating 30 significantly upregulated miRNAs (red dots, top 5 miRNAs are annotated: miR-31-5p, miR-215-3p, miR-215-5p, miR-30a-5p and miR-204-5p) and 15 downregulated miRNAs (blue dots, top five are annotated: miR-200-3p, miR-203-3p, miR-147b, miR-1247-5p and miR-552-5p) with fold change >1. **B** Heatmap of significantly up-regulated miRNAs, for which expression differed significantly between the obese individuals pre- and post-RYGB. **C** Heatmap of significantly downregulated miRNAs, for which expression differed significantly between the obese individuals pre- and post-RYGB. **D** IPA network of miRNAs for which expression differed by logFC >1 in the pre- and post-RYGB and their predicted target molecules.

Six months after RYGB, a total of 60 miRNAs (range −2.73 to 6.33 logFC) were changed significantly in the initially obese individuals. Weight loss up-regulated 38 miRNAs and downregulated 22 miRNAs significantly (Fig. 2A and Table S6). Expression levels of the significant up- and downregulated miRNAs in the comparison between obese individuals pre- and post-RYGB are illustrated in Fig. 2B and C, respectively. Differential expression patterns were observed between pre- and post-RYGB (Fig. 2B, C). It is apparent that patterns of miRNA expression are similar in both the post-RYGB and the non-obese Control groups, and differ from those in the obese individual pre-RYGB. Using the 45 miRNAs for which expression differed by logFC >1 between obese individuals pre-RYGB and following RYGB, IPA analysis yielded a network that predicted interactions between 33 molecules (Fig. 2D). Following weight loss by RYGB, *AGO2, DICCER1*, *DELTA133P53* and *CCNE1* show multiple new interactions in this network (Fig. 2D) when compared to the first network (Fig. 1D). Notably, newly observed interactions include that *AGO2* is targeted by miR-29a-3p, targets miR-29 and is associated with a further four network members (*DCP2, CALCOCO2, DELTA133P53* and miR-15a-5p). In turn, *DICER1* is targeted by *CLACOCO2* and targets miR-221. The regulatory molecules involved in CRC development, *DELTA133P53* and *CCNE1*, are also actors in this network. *DELTA133P53* is targeted by *JAK2 and SSB*; targets four network members (miR-15a-5p, miR-29, miR-29a-3p and miR-221); and is associated with a further three molecules (*AGO2, CCND1* and miR-9). In turn, *CCNE1* is targeted by *CCND1* and 15a-5p. The interplay between these molecules and miRNAs (Fig. 1D) is predicted to influence the development of cancer, GI disease, inflammatory disease and response, tumour morphology, cell death and survival, cellular growth and proliferation and cell-to-cell signalling and interaction (Figure S2 and Table S7).

**Fig. 3:**
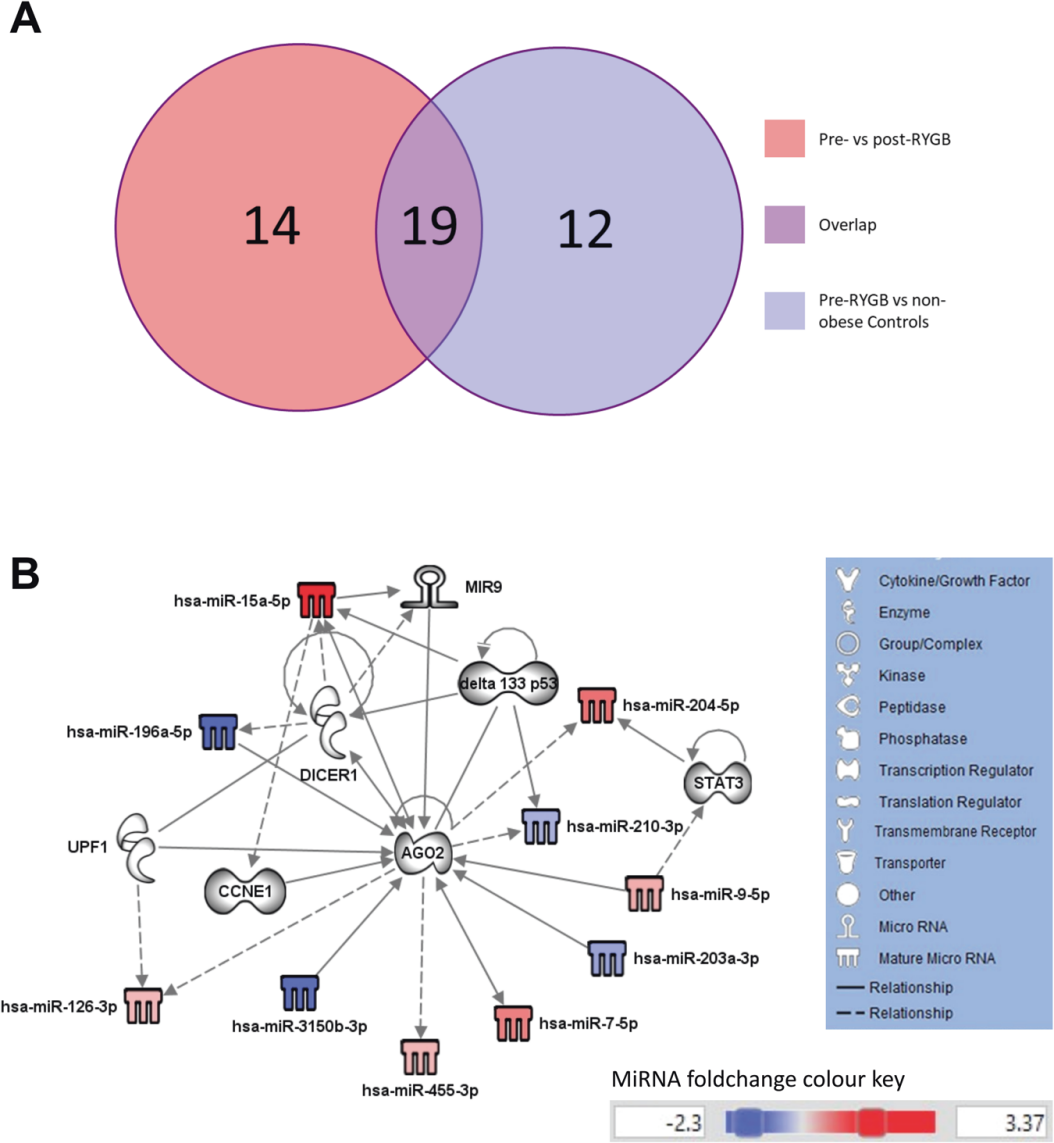
Predicted target molecules are implicated in obesity and influenced by weight loss. **A** Venn diagram showing the number of molecules (including miRNAs) identified in the comparison of obese patients pre- vs post-RYGB (total *n* = 33) and in the comparison of obese patients pre-RYGB vs non-obese Controls (total *n* = 31) and the number of overlapping molecules between the two comparison groups (total *n* = 19). **B** IPA Network of 19 overlapping miRNAs and predicted target molecules derived from miRNAs that were differentially expressed in patients with obesity vs non-obese Controls and in patients with obesity before and after RYGB.

To investigate which miRNAs, and predicted target molecules, are implicated in obesity and are changed in response to weight loss, we compared molecules from both networks (Figs. 1D and 2D) and found that 19 molecules were overlapping (Fig. 3A), of these 11 were miRNAs (Table S8). These miRNAs were then used to generate a biological network (Fig. 3B). Unsurprisingly, some were the same molecules implicated in miRNA biogenesis (*AGO2* and *DICER1*) and CRC development (*DELTA133P53*, *CCNE1* and STAT3) observed in the first two networks (Figs. 1D and 2D). *AGO2* emerged as a central player that is implicated in both obesity and in weight loss and which shows the highest number (*n* = 15) of interactions. It is targeted by 7 network members (*CCNE1, UPF1*, miR-9, miR-9-5p, miR-196a-5p, miR-203a-3p and miR-3150b-3p), targets four network members (miR-126-3p, miR-204-5p, miR-210-3p, miR-455-3p) and is associated with a further 4 molecules in this network (*DELTA133P53, DICER1*, miR-7-5p and miR-15a-5p).

### Comparison of genome-wide miRNA expression patterns between the non-obese controls and the obese participants post-RYGB

When comparing genome-wide miRNA expression patterns between the non-obese Controls and the obese participants post-RYGB, no significant differences were observed (Table S9).

### Expression of oncogene miR-31 and TSG miR-215 is elevated in rectal tissue from obese individuals and downregulated following RYGB

**Fig. 4:**
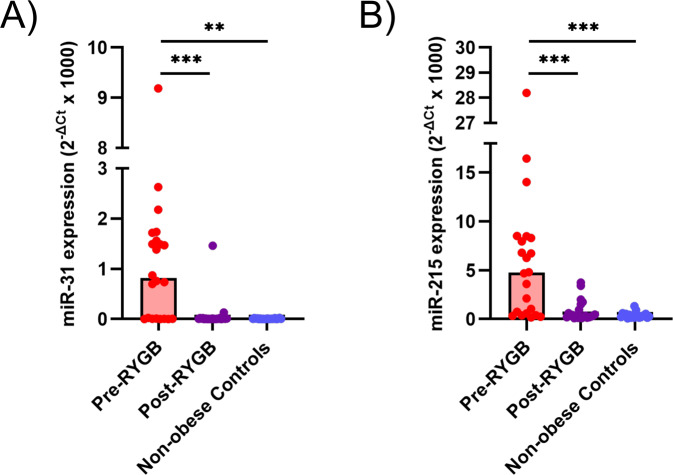
MiRNA expression determined by qPCR in the rectal mucosa of initially obese individuals pre- and post-RYGB and in non-obese Controls. **A** MiR-31 expression. **B** MiR-215 expression.

To validate the observation of differential miRNA expression obtained following NGS, qPCR was used to measure the expression of a panel of eight miRNAs. This confirmed the differential expression of miR-31 and miR-215 in obese individuals before and after RYGB (Figs. 4 and S3). The remaining six miRNAs (miR-204, miR-671, miR-892c, miR-1247, miR3196 and miR-4516) were unrelated to changes of adiposity, or their role is unknown, and will not be discussed further (Table S10).

## Discussion

This study of miRNA expression in the rectal mucosa of obese individuals before and after RYGB and in non-obese Controls revealed an obesity-related pattern of miRNA expression that is abrogated following weight loss induced by bariatric surgery.

NGS revealed a total of 1,654 miRNAs that were expressed in the human rectal mucosa. Of these, 112 miRNAs were differentially expressed in obese individuals compared with the non-obese Controls. Additionally, a total of 60 miRNAs changed expression in initially obese individuals at 6 months after RYGB. No significant differences in miRNA expression patterns were observed when comparing the non-obese Controls and the obese participants post-RYGB. Validation by qPCR confirmed our observation that obesity was associated with significant overexpression of miR-31 and miR-215 in the rectal mucosa when compared with non-obese Controls. In addition, RYGB resulting in mean 27 kg body weight loss at 6 months follow-up led to significant falls in expression of both miR-31 and miR-215. Expression levels for these miRNAs after RYGB were similar to those observed in the non-obese Controls.

Focussing on adiposity, inflammation and CRC risk, we used IPA to investigate the potential functional consequences of those miRNAs showing the greatest expression changes. In the comparison of obese individuals before RYGB with non-obese Controls, IPA predicted multiple interactions between miRNAs and molecules required for miRNA biogenesis (*AGO2* and *DICER1*) and those implicated in CRC development (*DELTA133P53*, *CCNE1* and *STAT3*) (Fig. 1D). Although the roles of *DELTA133P53* and *CCNE1* in obesity remain to be discovered, their roles in CRC are well characterised. Evidence from the IPA obtained here, suggests that *DELTA133P53* and *CCNE1* play roles in body fatness and, because they are known to have a role in CRC [21], they may mediate the relationship between body fatness and CRC. *DELTA133P53* is more highly expressed in cancer tissue and has been implicated in tumour development [21]. *DELTA133P53* alters promoter selectivity and *P53* transcriptional activity, by binding to the promoters of repair-related genes including *LIG4, RAD51* and *RAD52*, which then promotes cell survival via induction of DNA repair and angiogenesis. *DELTA133P53* also regulates gene expression (e.g. increasing *BCL2L* expression) resulting in inhibition of apoptosis and upregulation of the immune response to tumour cell proliferation [18, 21]. *CCNE1* (encoding the protein cyclin E) is overexpressed in multiple GI cancers, including CRC, leading to chromosome instability and contributing to tumour development [17]. Cyclin E is also involved in the initiation of DNA replication and centrosome duplication. In normal cells, cyclin E becomes phosphorylated and is degraded to allow cell cycle progression, whereas excessive cyclin E expression drives premature DNA replication leading to tumourigenesis [23].

When comparing obese individuals pre-RYGB and following RYGB, IPA predicted similar interactions to the first network (Fig. 1D), with the exception of *STAT3* (Fig. 2D). This suggests that weight loss by RYGB was insufficient to induce *STAT3* involvement. At 6 months post-RYGB, individuals had not yet achieved the BMI of non-obese Controls and a bigger difference in adiposity may be needed to affect *STAT3*. *STAT3* mediates the differentiation of preadipocytes to mature adipocytes, augmenting lipid accumulation, whereas *STAT3* inhibition stops preadipocyte differentiation [24]. Additionally, *STAT3* is a transcription factor for oncogenic signalling in CRC that is associated with poor prognosis and lymph node involvement [19, 22] and has been linked to adverse inflammatory response and poor survival in CRC patients [20]. *STAT3* transduces signals from extracellular stimuli to several interferons (IFN- α, -β, and - ɣ) and cytokines (gp130, IL-2, −6, −12, −15, −21 and −23) and thereby directs cell transformation and tumourigenesis [19].

**Fig. 5:**
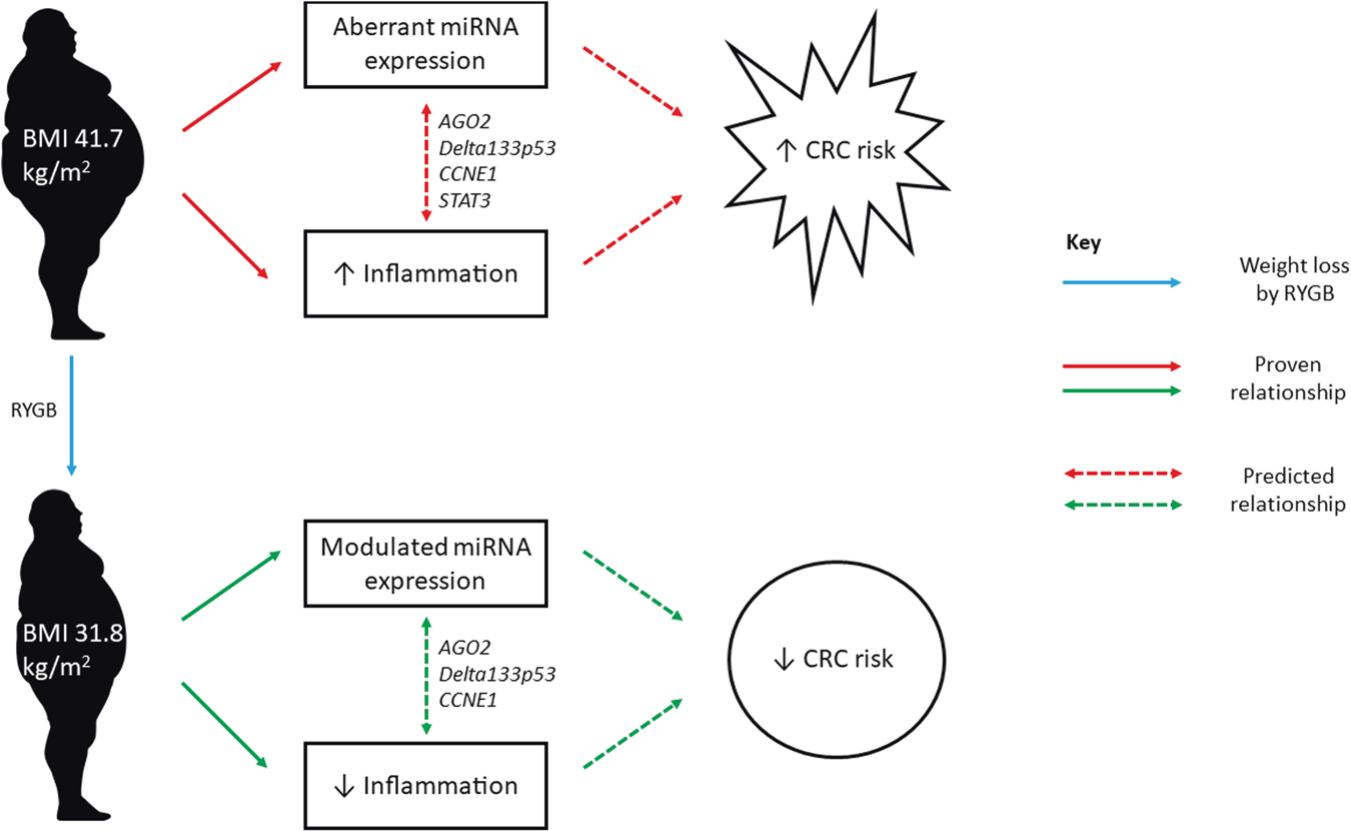
Cross-talk between adiposity, weight loss and CRC risk. *AGO2* communicates with *DELTA133P53*, *CCNE1* and *STAT3* in obese patients to potentially mediate increased CRC risk via increased inflammation. *AGO2* communicates with *DELTA133P53* and *CCNE1* in non-obese patients following RYGB to potentially mediate reduced CRC risk via reduced inflammation.

*AGO2* emerged as a central regulatory molecule in the network containing miRNAs implicated in both obesity and weight loss **(**Fig. 3A**)**. Critically, AGO2 regulates both energy production and energy utilisation. In mice, Ago2 causes metabolic disruption through regulating the expression of specific miRNAs including miR-802, miR-103/107, and miR-148a/152, and, at the same time, suppresses the expression of genes regulating glucose and lipid metabolism, including *Hnf1β*, *Cav1*, and *Ampka1* [25]. Although PPARα did not feature as a key molecule in our pathway analyses, recent studies carried out in the liver suggest that Ago2 function is intrinsically associated with PPARα and, consequently, with mitochondrial energy metabolism that may contribute to obesity-associated pathophysiology [26]. In addition, in mice, intestinal knockout of PPARα promotes colon carcinogenesis by increasing DNMT1-mediated methylation of P21 and PRMT6-mediated methylation of p27 [27]. Potential interactions between AGO2 and PPARα in the human colorectal epithelium remain to be investigated. However, as the central molecule in our network (Fig. 3B), communicating with *DELTA133P53*, *CCNE1* and *STAT3*, *AGO2* may mediate cross-talk between adiposity, weight loss and CRC risk with inflammation as the potential causal pathway (Fig. 5).

Obesity is associated with abnormal patterns of miRNA expression that occur in response to, or are driven by, chronic inflammation in obese adipose tissue [9]. Patterns of miRNA expression may be “normalised” to some extent following significant and sustained weight loss as a result of bariatric surgery [28–35]. However, most of this evidence has been obtained from measurements made in blood, one study has reported miRNA expression in adipose tissue [28] and there are no comparable data from the rectal mucosa. In the present study, oncogenic miR-31 and tumour suppressive miR-215 were aberrantly expressed in obese individuals, before RYGB, and their expression levels were normalised to those comparable with non-obese Controls following weight loss surgery. Although miR-31 and miR-215 were not identified in the IPA-predicted networks here (Figs. 1D, 2D and 3A), other studies have found that these two miRNAs regulate some of the molecules in our networks. The isoform miR-31-P targets and represses *DICER* by translational repression, whereas miR-31-M targets and represses *CSBPα, STK40* and *E2F2*, in HCT116 colon cancer cells, suggesting that specific genes regulated by miR-31 might be isoform dependent [36]. MiR-215 also targets and downregulates *DICER1* in HCT-116 cells [37]. In addition, miR-31 regulates cross-talk between NF-κB and *STAT3*, which was associated with the progression of late-stage tumour development in a murine model of CRC [38]. MiR-215 targets and inhibits XIAP expression, a suppressor of apoptosis, which may facilitate CASP3 and CASP9 activity and subsequently induce apoptosis of human CRC cells [39]. The STAT3 signalling pathway directly targets miR-31 and upregulates its expression in mouse intestinal epithelial cells, with downstream targets including the WNT, BMP and TGFβ pathways, and promotes colorectal tumorigenesis [40]. Studies in other GI cancers reveal an interaction between miR-31 and STAT3. For example, inhibition of STAT3 signalling reduced miR-31 expression and cell proliferation in bile duct cancer cell lines (HuCCT-1 and TFK-1) [41]. In the CRC cell line SW480, *STAT3* knockdown resulted in miR-215 downregulation [42]. In conclusion, the bidirectional interaction between *STAT3* and the miRNAs miR-31 and miR-215 may be an important pathway through which obesity increases CRC risk.

### Excess adiposity dysregulates expression of miR-31 and miR-215

MiR-31 is an oncogene that is upregulated during CRC [8] and also promotes adipogenesis [11]. Given the link between adiposity and CRC risk, our finding of increased miR-31 expression in the human rectal mucosa of obese individuals pre-RYGB (when compared with the non-obese Controls) is consistent with the role of miR-31 as an oncogene. This is in line with findings from Kurylowicz and colleagues [43] and Liao and colleagues [28] who reported increased miR-31 expression in both visceral and subcutaneous fat of obese individuals compared with non-obese controls. Our study is the first to report obesity-related increased expression of miR-31 in the human rectal mucosa.

Given the link between adiposity and CRC risk and the role of miR-215 as a TSG in CRC [44], the finding of increased miR-215 expression in the rectal mucosa of obese individuals is unexpected. However, this finding is in line with findings from Kurylowicz and colleagues [43] who also observed increased expression of miR-215 in obese, compared with normal weight, adults. Both findings may be unrelated to pathology. Nonetheless, because miR-215 has an established role in obesity [45] and in colorectal tumorigenesis [44], this finding requires further investigation.

### Significant and sustained weight loss by RYGB in obese individuals normalises miR-31 and miR-215 expression to expression levels comparable to that of non-obese Controls

Given the link between adiposity and CRC risk, the finding of reduced miR-31 expression in the rectal mucosa following surgically-induced weight loss is consistent with the role of miR-31 as an oncogene [46] and may indicate lower CRC risk. Although Kurylowicz and colleagues [43] reported higher miR-215 expression in adipose tissue from obese individuals, they did not observe any change in expression following weight loss surgery. In contrast, in the present study, miR-215 expression was normalised 6 months after RYGB. These findings indicate that the epigenetic landscape of the human rectal mucosa is plastic and responds to changes in adiposity that, we hypothesise, may modulate tumorigenesis.

The main strength of this study was the measurement of miRNA expression in the tissue of interest, namely the rectal mucosa. To date, this is the first human study to examine the effects of adiposity, and of weight loss, on miRNA expression in the rectal mucosa. A further strength is the use of paired rectal mucosal biopsies from the same anatomical site in the same individuals pre- and post-RYGB. In addition, we recruited obese patients and the control group from the same geographical area which ensured that individuals had similar environmental exposures. Although our participants were followed-up at six months post-RYGB when they had lost, on average, 27.1 kg body weight, they had not yet achieved the BMI of the non-obese control group (31.8 and 25.4 kg/m^2^, respectively). A final limitation is the relatively small numbers of individuals in both the obese (*n* = 22) and non-obese control (*n* = 20) groups. Future studies should enrol larger numbers of individuals and follow-up for longer until BMI is in the non-obese range.

## Conclusion

This study provides the first evidence that miRNA expression in the human rectal mucosa is altered in obesity and that miRNA expression may be normalised following deliberate, and sustained, weight loss after bariatric surgery. In particular, expression of the oncogene miR-31 and the TSG miR-215 responds to changes in adiposity. Such weight loss also improves systemic markers of inflammation and of metabolic stress that may indicate reduced CRC risk. We hypothesise that these miRNAs mediate the effects of adiposity on CRC risk and this should be tested in future studies.

## Supplementary information

Supplementary file

Supplementary table 1

Supplementary table 2

Supplementary table 4

Supplementary table 5

Supplementary table 6

Supplementary table 9

## Data Availability

All data relevant to the study are included in the article or uploaded as online supplementary information.
