# Supplementary material for: Obesity and Roux-en-Y gastric bypass drive changes in miR-31 and miR-215 expression in the human rectal mucosa
Source: Int J Obes (Lond). 2021 Oct 29;46(2):333–41. doi: 10.1038/s41366-021-01005-y (PMC8794786; doi:10.1038/s41366-021-01005-y)
Supplement: Supplementary file 1 — Supplementary file [file 41366_2021_1005_MOESM1_ESM.pdf]

Supplementary figures:

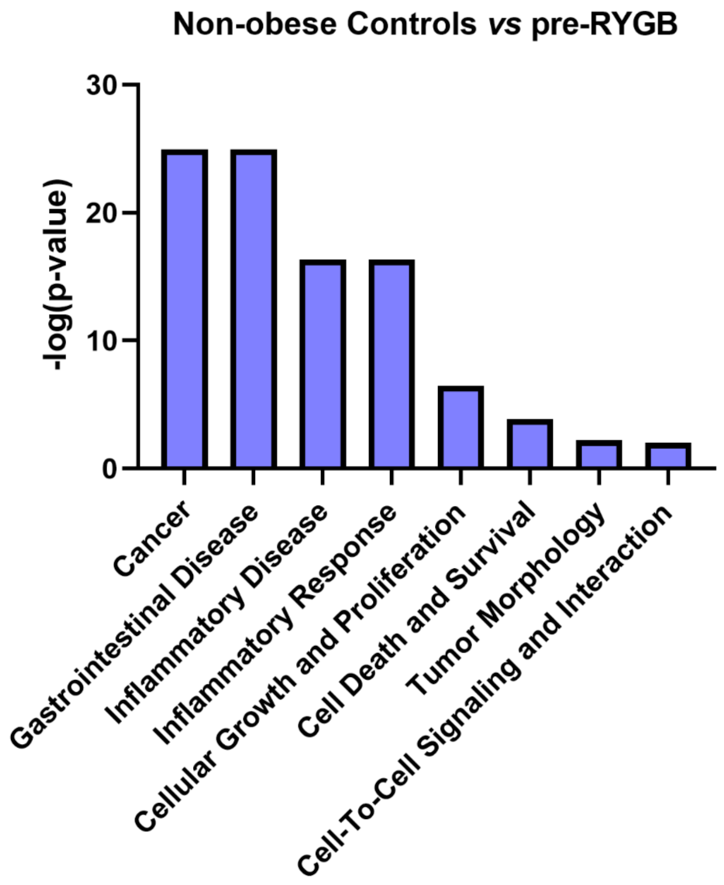

Figure S1: Significant IPA predicted interplay in diseases between miRNAs and molecules occurring in network number 1 (Figure 1D) when comparing non-obese Controls with obese individuals before RYGB.

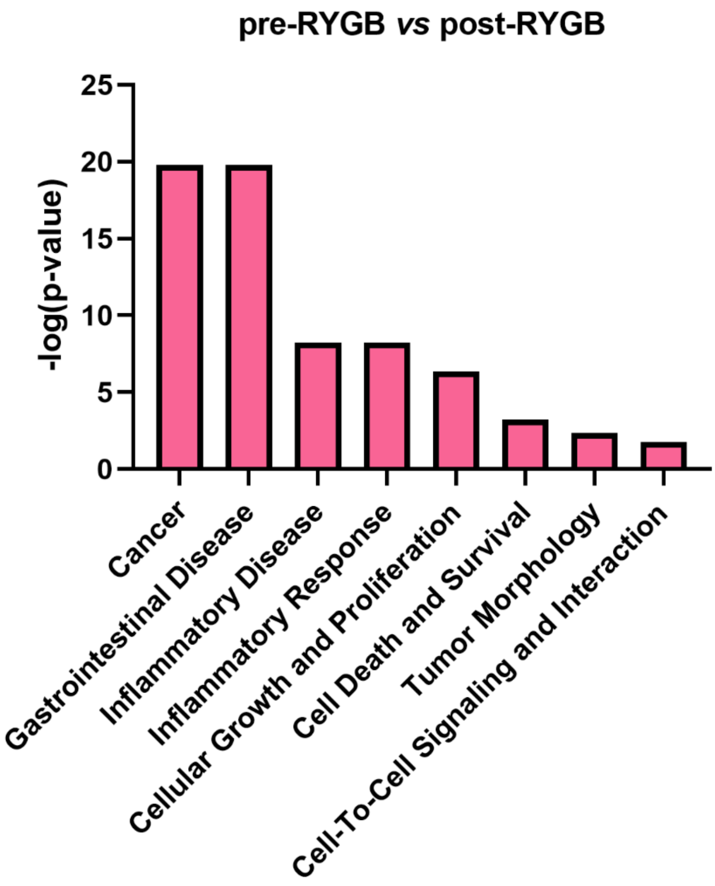

Figure S2: Significant IPA predicted interplay in diseases between miRNAs and molecules occurring in network number 2 (Figure 2D) when comparing obese patients pre- vs post-RYGB.

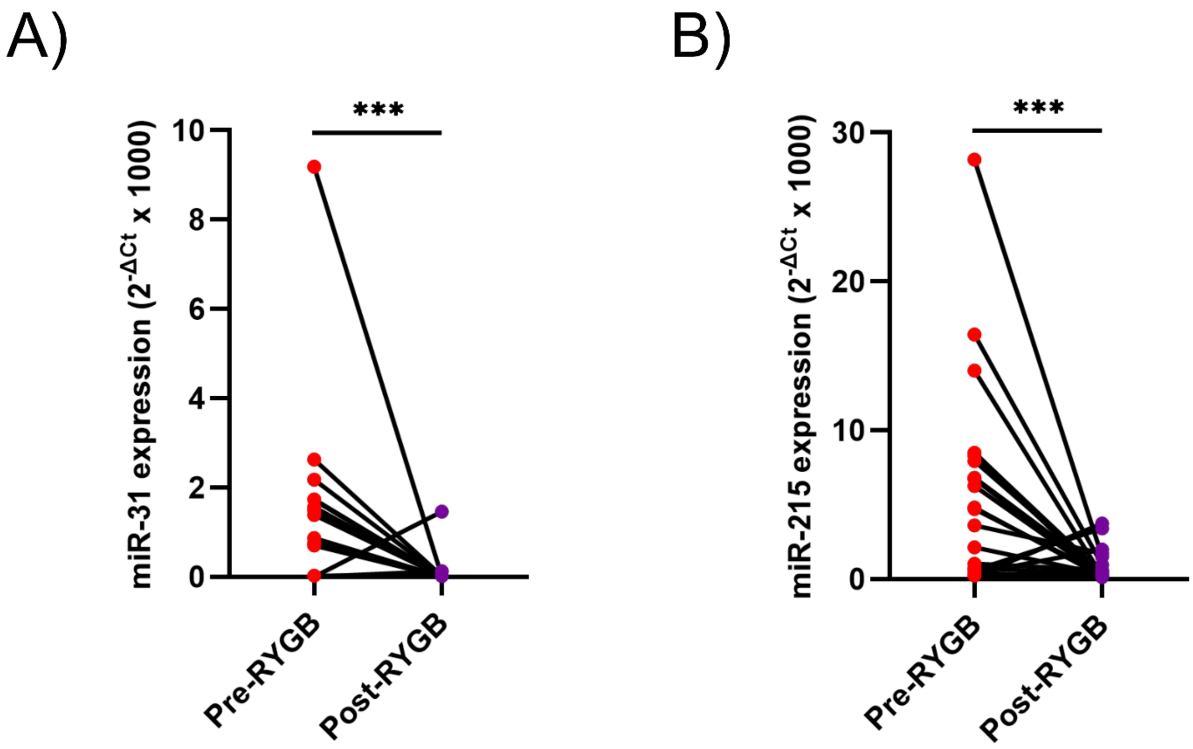

Figure S3: Inter-individual change in miRNA expression in the rectal mucosa of participants pre- and post-RYGB A) miR-31 B) miR-215.

Supplementary tables:

Table S3: Mean raw and normalised miRNA counts for pre- and post-RYGB patients and non-obese Controls.

	Pre-RYGB patients	Post-RYGB patients	Non-obese Controls
**Raw counts**	206515.5	195584.6923	124993.0909
**Normalised counts**	150204.25	149292.9989	158932.0198

Table S7: Significant IPA predicted interplay in diseases between miRNAs and molecules occurring in network number 2 (Figure 2D) when comparing obese patients pre- vs post-RYGB.

Disease	p-value	minus log pvalue	Molecules
Cancer	1.54E-20	-19.8125	let-7a-3p, let-7a-5p, miR-125b-5p, miR-126a-3p, miR-126a-5p, miR-129-5p, miR-132-3p, miR-148a-3p, miR-15b-3p, miR-16-5p, miR-181a-1-3p, miR-185-5p, miR-191-5p, miR-192-5p, miR-194-5p, miR-196a-5p, miR-200a-5p, miR-200b-3p, miR-203a-3p, miR-204-5p, miR-210-3p, miR-223-3p, miR-29b-3p, miR-30a-3p, miR-30c-5p, miR-31-5p, miR-335-3p, miR-338-3p, miR-342-3p, miR-421-3p, miR-450a-5p, miR-452-5p, miR-455-3p, miR-652-3p, miR-671-5p, miR-7a-5p, miR-874-3p, miR-9-5p
Gastrointestinal Disease	1.54E-20	-19.8125	let-7a-5p, miR-125b-5p, miR-126a-3p, miR-126a-5p, miR-129-5p, miR-148a-3p, miR-16-5p, miR-181a-1-3p, miR-185-5p, miR-191-5p, miR-192-5p, miR-196a-5p, miR-200a-5p, miR-200b-3p, miR-203a-3p, miR-204-5p, miR-210-3p, miR-223-3p, miR-28-3p, miR-29b-3p, miR-30a-3p, miR-30c-5p, miR-31-5p, miR-421-3p, miR-450a-5p, miR-452-5p, miR-455-3p, miR-652-3p, miR-671-5p, miR-7a-5p, miR-874-3p, miR-9-5p
Inflammatory Disease	5.98E-09	-8.2233	let-7a-5p, miR-125b-5p, miR-126a-3p, miR-129-5p, miR-148a-3p, miR-16-5p, miR-181a-1-3p, miR-185-5p, miR-192-5p, miR-196a-5p, miR-200b-3p, miR-203a-3p, miR-210-3p, miR-223-3p, miR-29b-3p, miR-30a-3p, miR-30c-5p, miR-342-3p, miR-671-5p
Inflammatory Response	5.98E-09	-8.2233	let-7a-5p, miR-125b-5p, miR-148a-3p, miR-16-5p, miR-185-5p, miR-192-5p, miR-200b-3p, miR-210-3p, miR-223-3p, miR-30c-5p, miR-342-3p, miR-671-5p
Cellular Growth and Proliferation	4.23E-07	-6.37366	let-7a-5p, miR-125b-5p, miR-126a-5p, miR-129-5p, miR-132-3p, miR-148a-3p, miR-16-5p, miR-185-5p, miR-192-5p, miR-194-5p, miR-200a-5p, miR-200b-3p, miR-203a-3p, miR-204-5p, miR-223-3p, miR-29b-3p, miR-30a-3p, miR-31-5p, miR-342-3p, miR-7a-5p, miR-874-3p
Cell Death and Survival	0.000569	-3.24489	let-7a-5p, miR-125b-5p, miR-129-5p, miR-148a-3p, miR-16-5p, miR-185-5p, miR-194-5p, miR-200b-3p, miR-203a-3p, miR-204-5p, miR-210-3p, miR-223-3p, miR-29b-3p, miR-455-3p, miR-7a-5p
Tumor Morphology	0.00433	-2.36351	miR-125b-5p, miR-16-5p, miR-200b-3p
Cell-To-Cell Signaling and Interaction	0.0172	-1.76447	miR-16-5p

Table S8: Overlapping molecules between the pre- vs post-RYGB network and the pre-RYBG vs non-obese Controls network from which the overlapping miRNAs were used to generate an additional network

Molecules
miR-126a-3p
miR-16-5p
miR-196a-5p
miR-203a-3p
miR-204-5p
miR-210-3p
miR-3150b-3p
miR-455-3p
miR-7a-5p
mir-9
miR-9-5p

Table S10: MiRNA expression (2-ΔCt x 1,000) determined by qPCR in the rectal mucosa of initially obese individuals pre- and post-RYGB and in non-obese Controls (NS: not significant; *: p-value ≤ 0.05; **: p-value ≤ 0.01)

miRNA	Obese pre-RYGB	Obese post-RYGB	Non-obese Controls	P-value; pre-RYGB *vs* Controls	P-value; pre- *vs* post-RYGB
miR-204	3.251229	2.037882	1.796871	NS	NS
miR-671	1.785026	1.556161	0.535563	NS	NS
miR-892	20.18802	11.68318	17.32017	NS	NS
miR-1247	1.806269	0.388467	0.289823	NS	NS
miR-3196	6.882955	5.672692	2.657616	*	*
miR-4516	680.5764	683.1289	257.739	**	NS
